# Rapid detection of talcum powder in tea using FT-IR spectroscopy coupled with chemometrics

**DOI:** 10.1038/srep30313

**Published:** 2016-07-29

**Authors:** Xiaoli Li, Yuying Zhang, Yong He

**Affiliations:** 1 College of Biosystems Engineering and Food Science, Zhejiang University, 866 Yuhangtang Road, Hangzhou 310058, China

## Abstract

This paper investigated the feasibility of Fourier transform infrared transmission (FT-IR) spectroscopy to detect talcum powder illegally added in tea based on chemometric methods. Firstly, 210 samples of tea powder with 13 dose levels of talcum powder were prepared for FT-IR spectra acquirement. In order to highlight the slight variations in FT-IR spectra, smoothing, normalize and standard normal variate (SNV) were employed to preprocess the raw spectra. Among them, SNV preprocessing had the best performance with high correlation of prediction (R_P_ = 0.948) and low root mean square error of prediction (RMSEP = 0.108) of partial least squares (PLS) model. Then 18 characteristic wavenumbers were selected based on a hybrid of backward interval partial least squares (biPLS) regression, competitive adaptive reweighted sampling (CARS) algorithm and successive projections algorithm (SPA). These characteristic wavenumbers only accounted for 0.64% of the full wavenumbers. Following that, 18 characteristic wavenumbers were used to build linear and nonlinear determination models by PLS regression and extreme learning machine (ELM), respectively. The optimal model with R_P_ = 0.963 and RMSEP = 0.137 was achieved by ELM algorithm. These results demonstrated that FT-IR spectroscopy with chemometrics could be used successfully to detect talcum powder in tea.

Tea is one of the most popular drinks throughout the world. Among all the organoleptic evaluation indexes of tea, color plays an especially important role in the quality and the market price of tea^1^,^2^. Therefore, some tea producers add talcum powder into the tea illegally to make it more attractive or conceal quality defects. Talcum powder, Mg_3_[Si_4_O_10_](OH)_2_, is an important industrial mineral and it is widely used in the manufacture of body and face powder^3^,^4^. Recently, many researches have proved that abusing talcum powder may increase the risk of cancer^5^,^6^. According to China food safety standards GB/T 14456.1^7^, it is banned to add any illegal adulterations in tea production. However, there is still no standard method for the detection of talcum powder in tea.

Unfortunately, research on talcum powder in tea is uncommon. Chen *et al.*^7^ and Xiang *et al.*^8^ investigated talcum powder in tea based on traditional chemical methods. In these researches, the existence of talcum powder in tea was arbitrarily inferred based on the existence of magnesium^7^,^8^. Actually, the tea itself has abundant magnesium, which is the integrant element of chlorophyll^9^. And soil metal pollution may also lead to the accumulation of magnesium in tea. So, the existence of magnesium cannot prove the existence of talcum powder. In addition, the traditional detection of adulterations is based on chemical analysis, which is trivial, expensive and time-consuming. Therefore, a rapid, simple and accurate detection method to evaluate the talcum powder added in tea is required.

Infrared (IR) spectroscopy has been known as a powerful tool for analysis of chemical constituents with specific frequency absorbance of functional groups. More complex structure leads to more absorption bands and more complex spectra. Rapid detection is the biggest advantage of IR spectroscopy. In 1977, Norris *et al.* first used near infrared (NIR) spectroscopy to study the protein in hard red winter wheat^10^. In recent decades, IR spectroscopy has been widely applied to analyze food quality^11^,^12^,^13^. Especially, an increasing number of researches on tea and adulterants in food are reported. Li *et al.* analyzed dry matter content of tea by near infrared (NIR) and mid-infrared (MIR) spectroscopy^14^. Botelho *et al.* applied MIR spectroscopy to detect five adulterants in raw milk^15^. However, there are still few studies on detection of adulterants in tea based on IR spectroscopy.

The IR spectra generally comprises a wide range of wavenumbers, some of which are useless or irrelevant information for modeling^16^. These interfered wavenumbers should be eliminated and the most relevant wavenumbers should be selected. Therefore, chemometric methods play a particularly important role in wavenumbers selection. In order to improve the accuracy of model and reduce the modeling time, more and more wavenumbers selection methods were reported nowadays, such as, regression coefficient analysis (RCA)^17^, Genetic algorithm (GA)^18^ and random forests (RF)^19^. In this research, a hybrid wavenumbers selection method, which combined backward interval partial least squares (biPLS) regression^20^, competitive adaptive reweighted sampling (CARS) algorithm^21^ and successive projections algorithm (SPA)^22^, was applied for IR spectral wavenumbers selection.

The aim of this study is to discriminate the tea samples added with talcum powder or not based on the IR spectra. Moreover, a hybrid method of biPLS, CARS and SPA was applied to select the most relevant wavenumbers and to build a model for quantitative detection of talcum powder in tea.

## Results and Discussion

### Overview of samples and spectral pre-treatment

**Figure 1:**
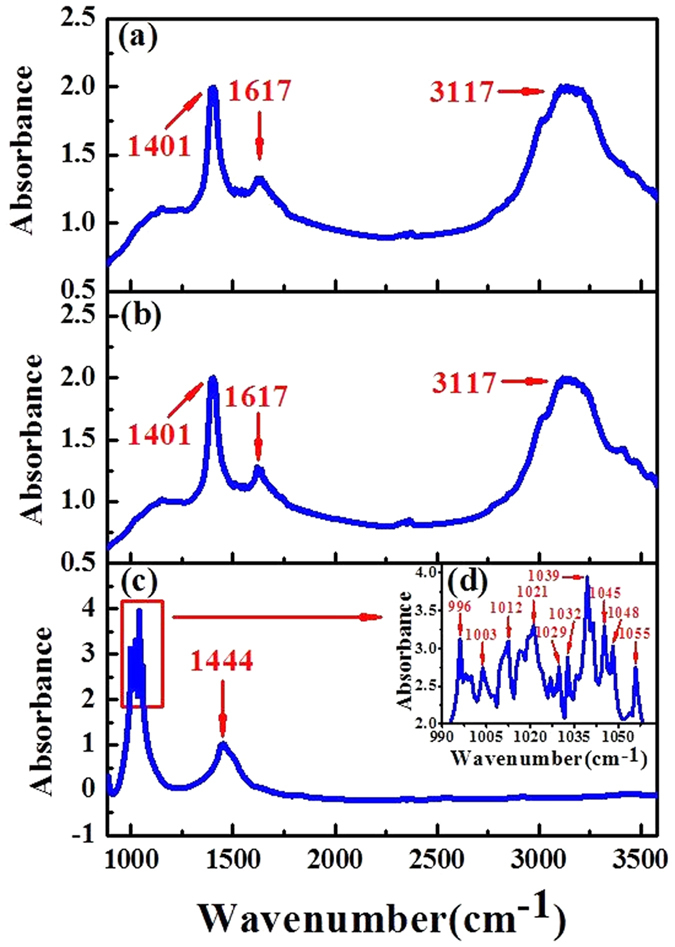
The average spectra of (a) pure tea sample, (b) tea sample with 1.50 mg/g talcum powder and (c) pure talcum powder including (d) the enlargement of the main peaks of pure talcum powder in the range of 990–1055 cm^−1^.

The average spectra of pure tea sample, tea sample with 1.50 mg/g talcum powder and pure talcum powder are demonstrated in Fig. 1(a–c), respectively. For the pure talcum powder spectrum, the main absorbance peaks were observed in the range of 990–1055 cm^−1^ (see enlarged peaks in Fig. 1(d)), which can be attributed to the Si-O-Si group^23^. In addition, another peak around 1444 cm^−1^ could be found in Fig. 1(c), which is related to Magnesite^24^. Comparing Fig. 1(a) with Fig. 1(b), the spectra of pure and adulterated samples have similar absorbance peaks at 1401 (related to N = N), 1617 (related to N = O) and 3117 cm^−1^ (related to C-H and O-H)^25^,^26^. Moreover, no distinct differences can be observed by naked eyes in Fig. 1(a,b). Therefore, it is in urgent need to build a model based on IR spectra for detection of talcum powder in tea.

**Figure 2:**
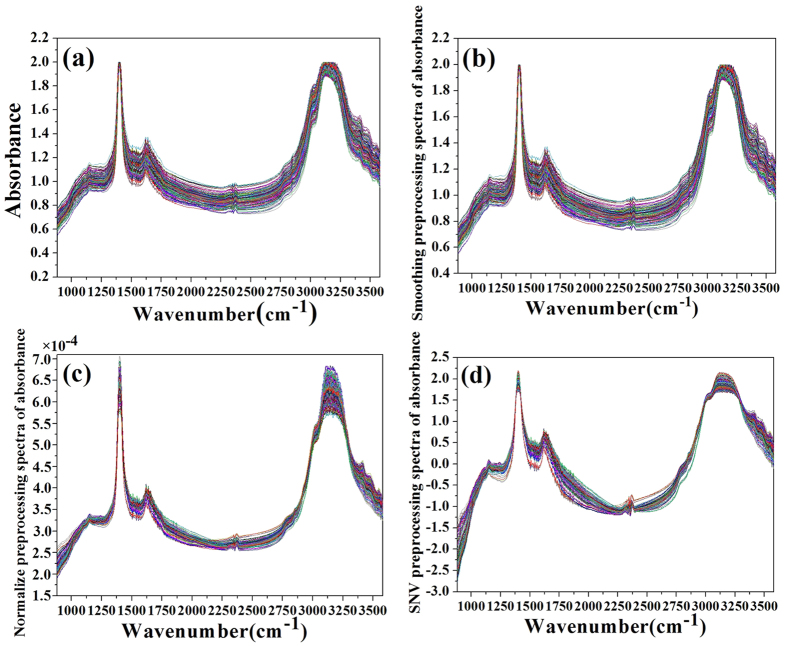
IR spectra of tea samples obtained from: (a) raw spectra, (b) smoothing preprocessing spectra, (c) normalize preprocessing spectra, (d) SNV preprocessing spectra.

In order to obtain a reliable, accurate and stable model, it is necessary to preprocess raw spectra before modeling, as shown in Fig. 2(a). Consequently, the spectral preprocessing methods of smoothing, normalize and standard normal variate (SNV) were applied comparatively in this research. And the spectra preprocessed using these methods were respectively presented in Fig. 2(b–d). To evaluate the effects of the spectra preprocessing, the data was used to build a PLS model, and the results were shown in Table 1. It could be found that the PLS model with SNV preprocessing (Model 4) had the best performance, which had a highest correlation of prediction (R_P_ = 0.948) and a lowest root-mean square error of prediction (RMSEP = 0.108). Therefore, the following chemometric analysis was based on the spectra after SNV preprocessing.

### Qualitative analysis

**Figure 3:**
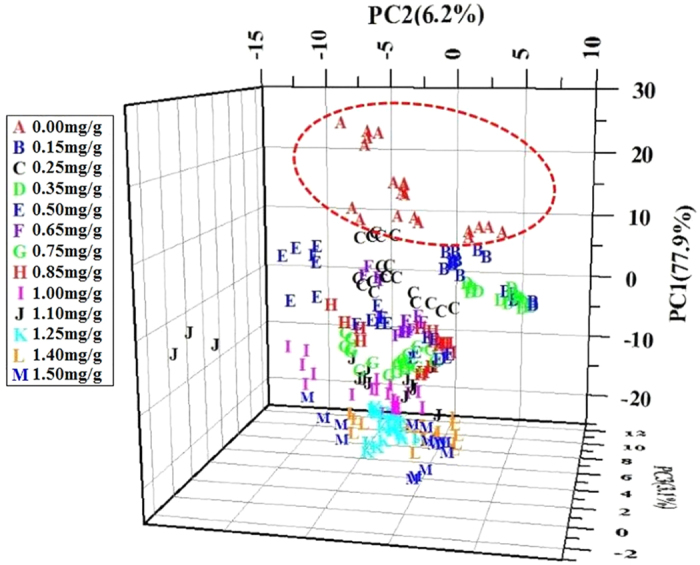
Scatter plot of three-dimensional PCs.

Before establishing quantitative model, principal component analysis (PCA) was used as a classification tool for analyzing tea sample with talcum powder or without. PCA is an unsupervised pattern recognition method, which can indicate the data trend of samples in visualizing dimension spaces^27^. To visualize the data trend of tea samples with different dose levels of talcum powder, a three-dimensional graph of tea samples using the first three principal components (PCs) were obtained, which was shown in Fig. 3. The PC1, PC2 and PC3 explained 77.9%, 6.2% and 3.1% of the variables, respectively. The total accumulative contribution rate of PC1, PC2 and PC3 accounted for 87.2%, which means the first three PCs could explain 87.2% of all information. It could be seen in Fig. 3 that pure tea samples and adulterated tea samples can be clearly separated. Whereas adulterated tea samples with different dose levels of talcum powder were clustered closely. It means PCA can be efficiently utilized for analyzing tea sample with talcum powder or without. However, it was not good at classifying adulterated tea samples with different dose levels of talcum powder. Therefore, quantitative analysis was adopted in the following studies.

### Quantitative analysis

#### Establishment of linear model

**Figure 4:**
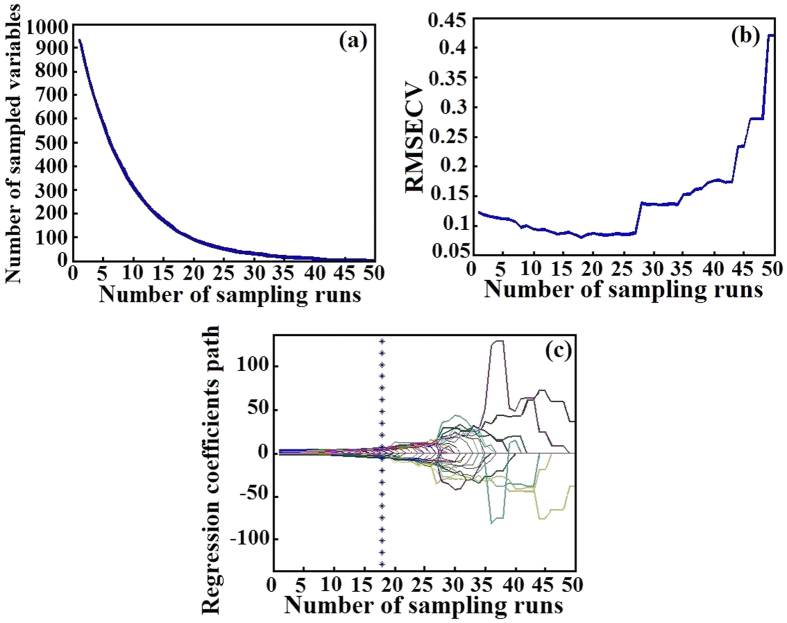
The process of CARS. (**a**) The changing trend of the number of sampled variables; (**b**) The changing trend of RMSECV; (**c**) The changing trend of regression coefficients of each variable.

In order to simplify the model and improve the performance of model, biPLS was first used to select the most relevant wavenumbers in this research. During the process of biPLS, firstly, the full spectrum from 881 to 3581 cm^−1^ were divided into 20 intervals with the same width manually. Then, leaving one subinterval out at a time, and the remaining subintervals were combined to build a PLS model. Repeating the previous step, several models with different number of intervals were achieved. Comparing the root-mean square error of cross validation (RMSECV) of each model, the one with lowest RMSECV was chosen as the best model. Thus, the intervals in the best model are the optimal ones. In this research, the final selected intervals were: 881–1080, 1182–1381, 1583–1682, 1783–1983, 2285–2384, 2686–2785 cm^−1^. Then these 936 wavenumbers were used to build a PLS model (Model 5), and the results were shown in Table 2. Comparing with Model 4, Model 5 was more simple, the wavenumbers of which was reduced from 2800 to 936, and its performance improved obviously with higher R_P_ = 0.949 and lower RMSEP = 0.107. However, the wavenumbers of Model 5 for modeling were still too much, which accounted for 33.46% of the full wavenumbers. So, CARS was used to reduce wavenumbers after biPLS processing. The process of CARS was shown in Fig. 4. It could be seen that the number of sample variables decreased fast in Fig. 4(a). And in Fig. 4(b), RMSECV declined at first, which indicates that some uninformative wavenumbers were eliminated. Then it increased rapidly due to removing some useful wavenumbers. Lines in Fig. 4(c) represented the coefficient of independent variables at different sampling runs. The vertical asterisk line was used to mark the optimal model with the lowest RMSECV. After the asterisk line, the RMSECV began to rise, which was attributed to the elimination of some effective wavenumbers. Finally, 111 wavenumbers, which accounted for 3.96% of the full wavenumbers, were selected by CARS to build a PLS model (Model 6). The results were shown in Table 2. It could be found that Model 6 had worse performance than both Model 4 and Model 5. But the gap between calibration and prediction in Model 6 became smaller than both Model 4 and Model 5, which means Model 6 had better stability and adaptability. Thus, CARS was effective for extraction of the optimal wavenumbers in this research.

**Figure 5:**
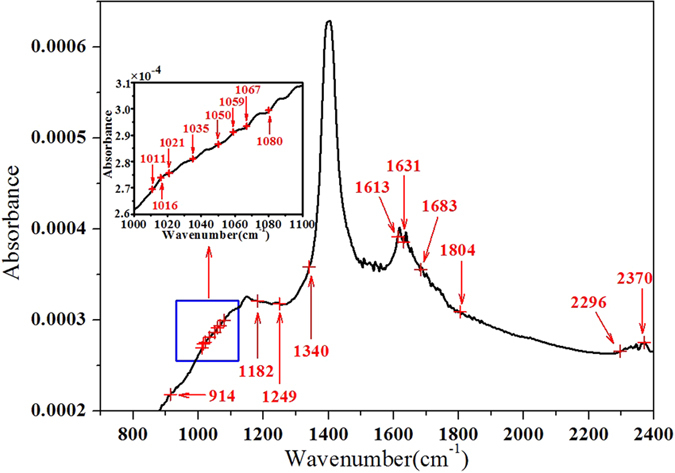
Selected wavenumbers by a hybrid of biPLS, CARS and SPA.

However, 111 wavenumbers were also a little more for establishing a simple and convenient model. Therefore, SPA was utilized on the basis of these 111 wavenumbers. It could be seen in Table 2 that 18 wavenumbers (914, 1011, 1016, 1021, 1035, 1050, 1059, 1067, 1080, 1182, 1249, 1340, 1613, 1631, 1683, 1804, 2296, 2370 cm^−1^) were selected as the most useful variables through SPA, which was only 0.64% of the full wavenumbers shown in Fig. 5. Combination of previous refs 24, 28, 29 and Fig. 1(c), 1016 (Si-O-Si), 1182 and 1249(CO-O), 1340 (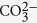), 1631 (OH) and 2296 (Si-H) cm^−1^ were the characteristic wavenumbers of talcum powder. Subsequently, these 18 wavenumbers were treated as input variables for establishing PLS model (Model 7), and the results were shown in Table 2. Comparing Model 5 and Model 6, the performance of Model 7 seemed slightly worse, but R_P_ = 0.931 and RMSEP = 0.124 of Model 7 were still acceptable. The most exciting thing was that the dimension of spectral variables reduced from 2800 to 18 characteristic wavenumbers, which resulted in a pretty simple linear model for the quantitative detection of talcum powder in tea. Meanwhile, these characteristic wavenumbers laid a solid foundation for revealing the detection mechanism of talcum powder in tea by IR spectroscopy. In conclusion, all these results indicated that a hybrid wavenumbers selection method, which combined biPLS regression, CARS algorithm and SPA, was effective for extracting the optimal wavenumbers from full spectrum. Meanwhile, it had good performance of the quantitative detection model of talcum powder in tea. More details of results of different wavenumbers selection methods were shown in Table 2.

### Establishment of nonlinear model

**Figure 6:**
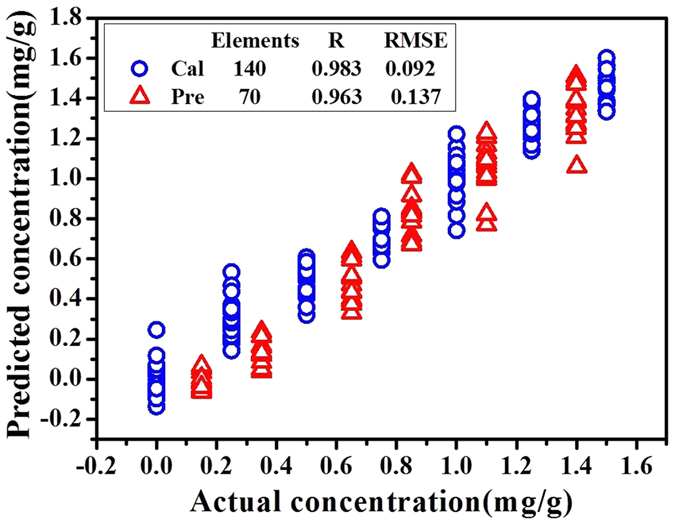
Scatter plots of actual vs. predicted concentration of ELM model.

In order to further improve the accuracy of the detection model, ELM was used to build a nonlinear model based on the above 18 characteristic wavenumbers. In the modeling, the activation function of ELM was set to “sigmoidal”. The number of hidden nodes, which was the only parameter needed to be determined manually, was varied from 1 to 80. The best model was achieved using 36 hidden nodes. The optimal results were obtained with R_P_ = 0.963 and RMSEP = 0.137 shown in Fig. 6. Compared with the prediction capacity of Model 7, ELM model had better prediction accuracy which indicated that ELM was more suitable than PLS for IR analysis in this research.

## Conclusion

In this research, the feasibility of FT-IR spectroscopy for the detection of talcum powder in tea was investigated. Comparing with previous studies, a hybrid of biPLS regression, CARS algorithm and SPA were first used to select optimal wavenumbers, which effectively extracted 18 characteristic wavenumbers from the full spectrum (3581 wavenumbers). Among these 18 wavenumbers, 1016(Si-O-Si), 1182 and 1249(CO-O), 1340 (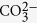), 1631(OH) and 2296(Si-H) cm^−1^ were the characteristic wavenumbers of talcum powder. And the linear and nonlinear detection models were built with these 18 characteristic wavenumbers by PLS and ELM, respectively. The optimal model was achieved with R_P_ = 0.963 and RMSEP = 0.137 based on ELM. All these results sufficiently indicated that FT-IR spectroscopy could be considered as a useful tool for rapid detection of talcum powder in tea.

## Materials and Methods

### Sample preparation

A set of 210 LongJing tea samples were purchased from a local market in Hangzhou, China. Talcum powder was purchased from Beijing Hagibis Technology Co.Ltd (Beijing, China). Firstly, Tea samples were ground into powder and filtered through a 60-mesh sieve. Then, 4.00 g pure sample was blended with 0.00 mg, 0.50 mg, 1.00 mg, 1.40 mg, 2.00 mg, 2.60 mg, 3.00 mg, 3.40 mg, 4.00 mg, 4.40 mg, 5.00 mg, 5.60 mg and 6.00 mg talcum powder. So the dose levels were 0.00 mg/g, 0.15 mg/g, 0.25 mg/g, 0.35 mg/g, 0.50 mg/g, 0.65 mg/g, 0.75 mg/g, 0.85 mg/g, 1.00 mg/g, 1.10 mg/g, 1.25 mg/g, 1.40 mg/g and 1.50 mg/g. Successively, 1.00 g blended sample was added into 49.00 g KBr medium, and mixed adequately for the following IR spectroscopy scanning. The 140 samples with concentrations of 0.00 mg/g, 0.25 mg/g, 0.50 mg/g, 0.75 mg/g, 1.00 mg/g, 1.25 mg/g and 1.50 mg/g were chosen as the calibration samples and the left 70 samples with concentrations of 0.15 mg/g, 0.35 mg/g, 0.65 mg/g, 0.85 mg/g, 1.10 mg/g and 1.40 mg/g were subsumed into the prediction set. Meanwhile, the calibration set was validated by full cross validation method. So the calibration, validation and prediction sets obtained 140, 140 and 70 samples respectively.

### Spectra collection

The IR spectra of samples were measured in the wavenumber range of 400–4000 cm^−1^ by a Jasco FT-IR-4100 spectrometer (Tokyo, Japan) coupled with a TGS detector and a ZnO crystal sampling accessory with transmission mode. The spectral resolution was 4 cm^−1^. Each spectrum was the average of 100 scans. During the whole experiment, the temperature was kept at about 25 °C and the humidity was kept at a stable level in the laboratory.

Raw spectra frequently contained noises besides sample information, so the first 500 and last 400 spectral data were deleted to remove noises, and the following analysis was based on the spectra in range of 881–3581 cm^−1^.

### Data analysis

#### Wavenumbers selection methods

In this research, a hybrid wavenumbers selection method, which combined backward interval partial least squares (biPLS) regression, competitive adaptive reweighted sampling (CARS) algorithm and successive projections algorithm (SPA), was applied for IR spectral wavenumbers selection. Backward interval partial least squares (biPLS) regression can extract the spectral wavenumbers highly related to the chemical structure^20^. CARS eliminates the wavenumbers with little or no effective information and retains effective wavenumbers according to the ‘‘survival of the fittest’’ principle^30^. Successive projections algorithm (SPA) has been proved to be a useful and effective tool for solving the collinearity problem with minimal redundancy^22^. Therefore, a hybrid of biPLS, CARS and SPA can combine the advantages of biPLS, CARS and SPA. It can achieve the objective to improve the stability of the prediction model and increase the interpretability of the relationship between the spectral response and chemical structure at the same time. Data analysis was performed on Matlab 7.0 (The Math Works, Natick, MA, USA).

#### Chemometric calibration method

Partial least squares (PLS) regression is a traditional technique for building linear models, which is able to not only extract principal component from both input and output data, but also determine the direction on which input and output data have the largest covariance^31^. In recent years, techniques for building nonlinear models coupled with spectroscopic techniques have also been successfully applied in many areas^32^,^33^,^34^. Extreme learning machine (ELM) is one of learning neural algorithms, which has been successfully applied in nonlinear regression problems^35^,^36^. Comparing with traditional learning algorithms, ELM not only reaches the smallest training error but also the least amount of output^37^. In this paper, ELM was used to build a nonlinear model and make a comparison with the linear PLS model. During modeling, full cross-validation was used to validate the quality of the models and to prevent over-fitting of the calibration. And the performance of models was evaluated by correlation and root-mean square error of calibration, cross validation and prediction (R_C_, R_CV_, R_P_, RMSEC, RMSECV, RMSEP)^38^,^39^.

## Additional Information

**How to cite this article**: Li, X. *et al.* Rapid detection of talcum powder in tea using FT-IR spectroscopy coupled with chemometrics. *Sci. Rep.* **6**, 30313; doi: 10.1038/srep30313 (2016).

## Figures and Tables

**Table 1:** Results for different spectral preprocessing methods by PLS.

Model No.	Preprocessing	R_C_	RMSEC	R_CV_	RMSECV	R_P_	RMSEP
Model 1	Raw data	0.959	0.141	0.958	0.144	0.933	0.123
Model 2	Smoothing	0.960	0.140	0.958	0.144	0.934	0.122
Model 3	Normalize	0.984	0.091	0.979	0.102	0.903	0.142
Model 4	SNV	0.972	0.118	0.969	0.125	0.948	0.108

**Table 2:** Results of different wavenumbers selection methods.

Model No.	Methods	Variable number	R_C_	RMSEC	R_CV_	RMSECV	R_P_	RMSEP
Model 4	None	2800	0.972	0.118	0.969	0.125	0.948	0.108
Model 5	biPLS	936	0.973	0.116	0.971	0.121	0.949	0.107
Model 6	BiPLS + CARS	111	0.965	0.131	0.962	0.136	0.942	0.114
Model 7	BiPLS + CARS + SPA	18	0.964	0.133	0.961	0.139	0.931	0.124
